# Social Media Analysis to Understand the Expected Benefits by Plant-Based Meat Alternatives Consumers

**DOI:** 10.3390/foods10123144

**Published:** 2021-12-18

**Authors:** Mirian Natali Blézins Moreira, Cássia Rita Pereira da Veiga, Zhaohui Su, Germano Glufke Reis, Lucilaine Maria Pascuci, Claudimar Pereira da Veiga

**Affiliations:** 1 Department of General and Applied Administration, School of Management, Federal University of Paraná—UFPR, Av. Lothtário Meissner, 632, Curitiba 80210-170, Brazil; mirian.ufpr@gmail.com (M.N.B.M.); cassia.veig@gmail.com (C.R.P.d.V.); glufkereis@ufpr.br (G.G.R.); 2 Mays Cancer Center, Center on Smart and Connected Health Technologies, School of Nursing, UT Health San Antonio, San Antonio, TX 78229, USA; szh@utexas.edu; 3 Department of Management, Center of Legal and Economic Sciences, Federal University of Espírito Santo—UFES, Av. Fernando Ferrari, 514-Goiabeiras, Vitória 29075-910, Brazil; lucilaine.pascuci@gmail.com

**Keywords:** foods, plant-based alternative meat, social media, classic marketing mix, manufactures, consumer

## Abstract

The plant-based alternative meat products market has attracted attention in recent years, as the demand for these products has grown worldwide. To meet the needs of this promising market, marketers must pay attention to the expected benefits of consumers and the insights that can be gleaned from comments posted on social media. This article proposed an investigation of the potential of the content analysis of comments posted on the Instagram social network of food companies that manufacture plant-based alternative meat products to understand the expected benefits by end consumers from the perspective of the classic marketing mix variables. The content posted voluntarily by consumers was organized into 13 categories of expected benefits analyzed within a proposal of evidence from the perspective of the marketing mix. The results showed that, among the insights obtained, 63% were related to the place variable, 21% to the product variable, 11% to the price variable, and 5% to the promotion variable. The insights reinforce the notion that marketing mix variables are crucial factors for companies to make products available in the right place, in the right quantity, and at a fair price, in addition to engaging with consumers through social media.

## 1. Introduction

The plant-based alternative meat products market has attracted attention in recent years [1,2] from researchers, the food industry, and public health policymakers [3] since the demand for these products has grown worldwide [4]. Plant-based products that resemble animal meat have stood out among alternatives to meat [5]. In the literature, several studies present the main definitions and differences between new plant-based alternative meat products about those based on original animal proteins [6]. To succeed in this new market, companies seek to maintain their products’ sensory, textural, visual, and taste components. These actions become important to assist consumers of traditional meat in the transition to a more plant-based diet [7,8].

Plant-based alternative meat products are part of this market and present a different proposal regarding traditional meat substitutes. Vegetable-based meat analogs aim to attract new consumers, including those who consume meat [9]. To attract new consumers in this expanding market, companies need to pay attention to classic marketing mix strategies to make products available in the right place, in the exact quantity, and at a fair price, in addition to engaging in assertive communication with their target audience [6].

The classic marketing mix, composed of the variable’s product, price, place, and promotion (4Ps) [10], is a well-documented strategy in the literature [11] and has been a challenge for many food companies [12] since their products are not sold directly to the final consumer [13]. Instead, these products are sold in retail through various marketing channels with many links before they reach the end consumer [14]. Promotion (communication) has been a powerful resource for bringing the manufacturer and the final consumer closer to each other [15]. To maintain the relationship with the end consumer and operate as experts for their customers, manufacturers must be familiar with the variables of the classic marketing mix to implement strategies to promote engagement and analyze the content posted by customers on social media.

Social media has been widely used as an effective tool that aids the strategic marketing direction of many manufacturing companies [16], especially in aspects related to customer engagement in management relationships and communication [17]. Some companies have developed social media marketing programs [18] to form a closer relationship with their customers [19]. The adoption of this new communication channel has resulted in many benefits, such as “increased revenue, cost reduction” [19] (p. 30), inspiration for advertising campaigns, and the development of new products [20], greater awareness of the company, and the brand, as well as better dissemination of products and services [21]. In the online consumer buying journey, it leaves digital footprints that can generate valuable benefits and data that help digital marketing managers in complex analytics to understand in-depth digital consumer buying behavior and develop more cost-effective marketing strategies for the target audience [22].

Social media can lead to greater potential benefits for some kinds of companies, such as those that manufacture niche products that use marketing channels with intermediaries. Considering that the creation of direct relationships between manufacturers and end consumers can generate long-term benefits for channel members [23], these companies can use the Internet and social media to minimize the restrictions imposed by physical distances, enabling them to boost the potential for expanding their markets [24]. Nonetheless, despite the potential benefits, little is known about how companies use social networking sites to achieve their goals [25], especially the use of social networks by vegetable-based meat analog manufacturers regarding the benefits sought by end consumers from the perspective of the marketing mix and the delivery of value to their end consumers [26].

Few works so far have empirically measured the potential use of analyzing content posted on social media so that manufacturers and end consumers in certain markets can form a closer relationship [27], as is the case of plant-based meat analogs [6]. The few existing works in the literature on the subject are exploratory and, therefore, only address broad themes addressed by consumers on social media, such as acceptance, legality, marketing, science and technology, and sustainability [28]. However, this provides little input regarding how companies can extract insights from posted content to deliver expected benefits to consumers. Furthermore, other research indicates that consumers expect to receive something tangible in return [19]. Nevertheless, there is little information on the benefits consumers seek in the exchange relationship they establish with the manufacturers. Therefore, the challenge lies in using social networks in practice to guide organizational strategies in meeting customer demands. Thus, further research on the subject can expand the possibilities of using social networks as a promising tool for formulating market strategies.

To bridge the research gap, this study aims to examine user-generated content (UGC) on social media to discover insights that help to understand the consumers’ expected benefits of plant-based meat alternatives. The UGC will be analyzed and classified from the classic marketing mix perspective: product, price, place, and promotion. In this article, we address these gaps by empirically examining the content posted on social media by end consumers in the light of the classic marketing mix to understand how the insights generated can help to classify the degree of importance of the marketing mix in the view of end consumers. The main contributions of this work are: (i) providing insights that can guide companies in making specific decisions about the marketing mix to meet the demands of their final consumers; (ii) and filling a gap in the literature on the applicability of using social networks to meet the benefits expected by consumers in the plant-based meat alternatives market.

## 2. Materials and Methods

### 2.1. Research Design

This is a qualitative study that adopted the content analysis approach [29] to identify insights from secondary data. The content analysis was based on UGC analysis [30]. This methodology allows us to understand the actual behavior of users [31], to the detriment of surveys that assess the preferences stated by respondents; that is, the findings are more accurate in the representation of users’ opinions compared to those collected from traditional data. As most production companies do not market their products directly to the final consumer, the experience of the marketing channel determines perceptions of brand image, the perceptions of the final consumer, and the effectiveness of the classic marketing mix strategy. For this purpose, consumer comments were collected from posts on the Instagram account of the Brazilian foodtech company “Fazenda Futuro” (https://fazendafuturo.io/, accessed on 24 November 2020) and the “Incrível Seara” line, belonging to the Brazilian company “Seara Alimentos” (https://www.lojaseara.com.br/categoria/incrivel, accessed on 24 November 2020).

**Figure 1:**
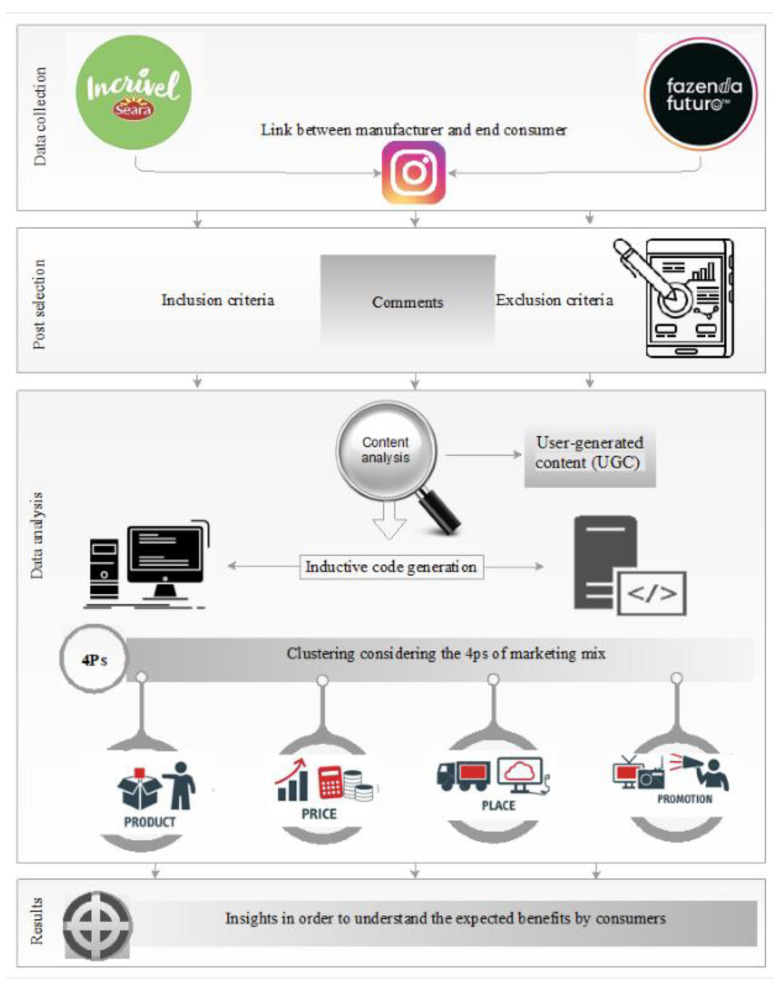
Research stages. Instagram—Incrível Seara and Fazenda Futuro.

Comments were collected from the Instagram social network from 26 November 2020 to 13 January 2021. The choice of this time interval was based on the high seasonality of the period, due to the growing trend in sales at the end of the year. The methodological procedures that were adopted are shown in Figure 1.

### 2.2. Selection Criteria

#### 2.2.1. Companies and Social Network

The two companies were selected for being pioneers in the vegetable meat segment in Brazil, a country recognized as a major producer of conventional meat of animal origin (Brazilian Association of Animal Protein [32,33]. Furthermore, both companies have social media profiles, an essential requirement for UGCs to be collected for analysis. Both companies have accounts on the social networks Facebook, YouTube, and Instagram. A prior consultation was conducted on the companies’ profiles on the three social networks to gauge the intensity of consumer comments on each one of them. The Instagram social network was selected for this study because the number of consumer comments was more expressive on this social network.

Fazenda Futuro has 260,000 followers on Instagram, for which it provides posts mainly related to its products. Its profile slogan on the page is “We make meat from plants just like other meat,” appealing to the similarity of its vegetable products to animal products (https://www.instagram.com/fazendafuturo/?hl=pt-br, accessed on 24 November 2020),. Incrível Seara, in turn, has 103,000 followers on the social network. In its profile presentation, the brand also refers to the flavor and texture of its products, comparing them with products based on animal meat. Their posts mainly show the products sold, but the brand also bets on using the image of famous personalities to promote its business (https://www.instagram.com/incrivelseara/?hl=pt-br, accessed on 24 November 2020).

#### 2.2.2. Posts and Comments

The inclusion and exclusion criteria adopted for the selection of the collected posts and comments are presented in Table 1.

Following the inclusion and exclusion criteria defined by the study, 635 comments were selected, with 312 collected from 20 posts on Fazenda Futuro and 323 from 18 posts on the Incredible Seara line. For Fazenda Futuro, the selected posts were made between 17 December 2020 and 13 January 2021, while the posts of the Incredible Seara line were made between 26 November 2020 and 12 January 2021. When the number of 300 comments collected from the posts of each of the companies was reached, the collection continued until the total textual comments of the valid posts were completed. The continuation of the collection was adopted so that the collection was not ended abruptly, which is why 635 comments were obtained.

### 2.3. Code Generation

The textual comments (UGC) were manually extracted from the selected companies’ Instagram profiles and were exported to a Microsoft Excel spreadsheet. Then, the collected data were subjected to content analysis, a technique that “explores the content of messages, addressing specific questions about ‘what,’ ‘who’ and ‘context’” and which can be used to analyze various sources, including websites [34] (p. 401). After an initial reading of all the comments by three researchers, initial codes were inductively generated to identify the expected benefits by consumers in their interaction with the manufacturers. Code generation enables an exploration of “the frequency and prominence of the message” [34] (p. 401). The codes were then grouped into broader themes according to the 4Ps of marketing: product, price, place (distribution), and promotion (axial coding). Finally, the results of previous analyses allowed the identification of insights that manufacturers could use to meet the expectations of their final consumers.

## 3. Analysis and Discussion of Results

Table 2 presents the user-generated content (UGC) in accordance with the criteria described in the Methods section of this study.

By the inclusion and exclusion criteria, 635 comments were selected of these, 300 generated insights regarding the expected benefits by consumers. This confirms that many consumers are willing to engage in a dialogue with manufacturers on social networks. This observation emphasizes that “in exchange for their time, endorsement and personal data, consumers expect something tangible” [19] (p. 31). This information is strategic for organizations since consumers can break off their relationship with the manufacturer if they do not feel that their efforts are reciprocated.

Some of the 300 comments contained two different relevant insights. In these cases, both insights were considered, which generated 326 initial codes during the inductive coding. The insights generated were grouped into 13 categories of expected benefits by consumers when interacting with the manufacturers on social networks. A minimum of three occurrences of the benefit was considered to form a category. Therefore, 298 coded comments were categorized in terms of the expected benefits by consumers.

Subsequently, the 13 categories that were generated were classified based on the classic marketing mix: product, price, place, and promotion, since the study’s proposal involved evaluating the total shopping experience of customers, not only requests for benefits concerning the products. Table 3 presents the content analysis results. This analysis included the 13 expected benefits by consumers and their relationship with the marketing mix. In this analysis, among the insights obtained, 63% (187) were related to issues regarding place, 21% (63) to products, 11% (33) to prices, and the remaining 5% (15) to promotion.

### 3.1. Place Variable (Distribution)

Contrary to the expectations of the authors of this study that most insights would be related to products, since manufacturers use social networks mainly to promote the items they sell, most of the insights generated refers to the strategic pillar of place. This finding demonstrates the strategic importance of understanding customer value from the perspective of the customers themselves. After all, “consumer values can be better measured directly by the consumers themselves (...)” [35] (p. 505). Table 4 details the comments classified into the place pillar. It is possible to note that the expected benefits by consumers are mainly related to (i) scope, (ii) distribution information, (iii) online channels, and (iv) assortment.

Coverage was the most desired benefit reported by consumers in the place variable. Many of the comments reflected customers’ desire to have physical access to the products advertised by the manufacturers. However, as cited in the examples below, some of these comments demonstrate that consumers were experiencing some degree of distress or frustration due to the difficulty involved in finding the desired products at the point of sale.

*“I would like to taste the chicken of the future. But unfortunately, I couldn’t find it anywhere in the city of Belo Horizonte.”*

*“Are they or are they not going to sell it in Ceará State? This is an outrage!!!”*

*“I don’t understand why you launch products but do not distribute them to all the regions of the country. It’s really hard to find your products!”*

The “information” attribute with regard to place was the second most desired benefit by consumers concerning the place variable. As evidenced below, this point portrays customer demand for more information related to place, such as (i) availability of products in certain locations or points of sale, (ii) expected date for the products to be available in a certain location, and (iii) availability of online sales channels or reasons for not covering certain geographic regions.

*“Can you help me by telling me the names of the stores where I can find the chicken of the future in the city of Belo Horizonte?”*

*“Why can’t I find it in the city of Vinhedo in São Paulo State?”*

*“Can’t I buy it on the site?”*

*“When will the products get to Rio Grande do Sul State?”*

The criterion used to differentiate the scope and distribution information categories was the affirmative or questioning tone of the comments. In the comments classified as coverage, customers showed dissatisfaction due to the products not being available in their regions after an effort to find them. In the comments classified as place information, customers seem to be at an early stage of searching for the products. Therefore, a lower level of dissatisfaction is perceived.

Comments classified as online channels demonstrate that consumers are interested in purchasing products through online channels because the products are not available in their own regions. However, it is important to emphasize the operational difficulty of using digital sales channels for such products considering the need for refrigeration, which would require important adaptations throughout the supply chain. Examples of comments classified as online channels are shown below:

*“Do you guys sell on the internet because where I live there’s nothing for sale!”*

*“There should be an option to buy through the website”*

*“Incredible Seara here in Betim-MG, I can’t find A THING!! They could sell directly on the website...”*

In the comments classified as assortment [36], it is observed that customers seek not only access to the manufacturers’ products but also the availability of a variety of products, as exemplified below:

*“Another product that I can’t find in the city of Porto Alegre is the sausages… I’ve given up on this brand because whenever I ask I always get the same answer: the list of points of sale here.......but none of them sell these products. Not even in November, as the company promised, did the sausages get here...”*

*“I can only find nuggets and shepherd’s pie in this line of products. For God’s sake, the partner markets only buy these. You can’t find veggie ham, meatballs or fish fingers. Nothing.”*

*“We don’t eat anything that’s available in this line of products because it’s all about animals. Just one thing, you need to provide the supermarkets with more varieties. I live in Juazeiro do Norte, Ceará, and I always buy from Assaí there, but I can only get kebabs and hamburgers. I want more he he he.”*

### 3.2. Product Variable

The second most frequently commented variable in the marketing mix was product, comprising 63 comments related to the products marketed by the manufacturers. Table 5 details the comments classified into the product variable. It is possible to note that the expected benefits by consumers are mainly related to (i) nutritional improvements in flavor or texture, (ii) nutritional information on the ingredients or preparation, (iii) new products, and (iv) information on availability.

Comments classified as nutritional, flavor, or texture improvements demonstrate consumers’ concern over the nutritional quality of the products they purchase and the taste of these products, as demonstrated below:

*“I think it’s amazing, but it could do with less salt!”*

*“Could be less sweet.”*

*“Incredible. I was impressed with everything, only the texture isn’t exactly the same for me, but still amazing!”*

*“I liked it, but it’s a bit too greasy and kind of nauseating TOO!”*

It is noteworthy that despite customers criticizing the taste/texture of the products and requesting improvements, many comments also demonstrate a positive shopping experience. However, as the objective of this study is to analyze comments from consumers who desire new benefits, the study does not include the attributes that customers considered as being in the products already.

The comments classified as nutrition, ingredients, or preparation information show the interest of end consumers in the characteristics intrinsic to the products and preparation process. Therefore, this type of comment can help manufacturers to formulate relevant content for their consumers. Considering that consumers may be uncertain about the health effects of meat substitutes (due to additives, artificiality, or possible lack of nutrients in these products [37]), manufacturers can use social media as both a channel to analyze doubts about providing information to customers in an attempt to generate greater perceived value. The following statements are examples of comments classified as nutrition, ingredients, or preparation information:

*“How can you make things with an Airfryer? I’ve looked for recipes, but I couldn’t find any.”*

*“What do you use to taste like cod, chicken, fish, ham, etc.?”*

*“Please tell me, do you need to fry the sausage before putting the pizza in the oven?”*

*“Do you have any videos about the production process??? I would like to know more about how it is made, it tastes wonderful! But are many chemical elements used to get the flavor and texture? @Fazendafuturo.”*

Comments classified as new products show end consumers’ interest in participating in the co-creation of the development of new products, as they presented information regarding products desired by customers. For example, it is important to report the interest in cheeses of vegetable origin, which were mentioned in eight comments. Although a vegetable cheese is not directly a meat substitute, consumers showed interest in co-creating this new item, demonstrating how social media can be strategic for manufacturers who intend to increase the list of products offered. The following examples exemplify comments classified as new products:

*“A kind of ham wouldn’t be amiss.”*

*“I have everything in stock from you guys. Please make cheese, too (images). I’ve never found a REALLY good one.”*

*“Wouldn’t it be nice if you guys launched a mozzarella and veg butter line?”*

Comments classified as product availability information refer to the availability of certain items. The search for information on availability reported in the product variable differs from the search for information on place reported in the distribution variable, as here, customers are not seeking information on the availability of products in a particular region or store, but in the continued production of a particular product. The comments that supported this insight can be seen below:

*“Do you still have this gammon, or was it seasonal?”*

*“Has product been discontinued?”*

*“Is there a shortage of sausages?”*

### 3.3. Price Variable

This research shows that the end consumers of vegetable-based meat analogs seek two types of benefits concerning the price of products through social media. Table 6 details the subgroups of comments classified into the price variable. It is possible to note that the expected benefits by consumers are mainly related to (i) price reduction and (ii) price information.

It is important to consider that value creation for customers is intrinsically related to the prices of the products on offer, as “value results from a trade-off between perceived costs and perceived benefits for the consumer” [35] (p. 505). As such costs are usually considered monetary [38], the prices charged represent financial resources that consumers will have to give up to obtain the benefits of the product. Thus, from an economic point of view, perceived value is related to the price that consumers agree to pay for a given offer [39]. Therefore, consumers consider price a critical factor in their purchasing decisions, albeit not the only one.

In the collected comments, it was noted that customers appreciate the manufacturers’ products, although price was a barrier to purchase intention. Considering that value can mean attractive products at affordable prices for certain consumers, if the company charges higher prices for its products than the value perceived by consumers, sales will be harmed [11]. Indeed, companies may lose the opportunity to generate experimentation with new customers due to the prices they charge.

*“I thought the idea was great...but FORTY-NINE REAIS?!??! No way I can pay that, right? I already think 20 for hamburgers and 17 for nuggets is expensive… I pay the price because I like these things, but there’s no way I can pay the price of the cod!”*

*“Good products, but very expensive. I can’t afford them.”*

*“Very Expensive!! For God’s sake, let’s rethink these prices”*

Some consumers also turn to social media for product pricing information. Customers want to know the prices charged and seek to understand the reason for these prices, which are considered high in the case of vegetable-based meat analogs. Comments classified as price information are detailed below.

*“How much does the sausage cost?!*

*“If it uses fewer resources, why is it more expensive than animal meat??”*

*“But why does this product have such a large price variation compared with the others in the line if they’re all made from vegetables?”*

### 3.4. Promotion Variable

The promotion variable was the one with the lowest number of spontaneous comments from end consumers. In general, Table 7 shows that these consumers use social networks to (i) request samples, gifts, or sponsorship, (ii) request greater disclosure of products, and (iii) express a desire for improvements to the website.

Comments related to requesting freebies, gifts, or sponsorship indicate the growing importance of digital influencers. Marketers increasingly realize that it is important to rely on opinion leaders and online personalities to promote their products, as the endorsement of digital influencers is often decisive for the acceptance of new products [40]. Of the 15 posts selected from the Incredible Seara profile, 3 included two digital influencers who are also celebrities outside the Internet. The comments also demonstrate the desire of some followers who identify with the product to become brand ambassadors. The comments transcribed below exemplify these findings:

*“Fazendafuturo, don’t be so boring and give me some freebies.”*

*“Please notice me and pamper me.”*

*“My dream is to be sponsored by Seara with this 100% vegetable line, you know...”*

Comments related to the product’s increased publicity suggest that meat replacement customers often champion an idea or cause. Thus, the consumption of this type of product may be linked to a greater objective: to transform the eating habits of a mostly carnivorous society. In fact, previous research has shown that the behavior of following more vegetable diets and reducing meat consumption may be motivated by concerns about health, sustainability, and ethics related to animals [6]. Thus, the search for greater dissemination of meat substitutes is potentially associated with the consumer’s desire to make this type of product known to others since meat is usually much more familiar to consumers than its substitutes [9]. The following transcripts detail examples of comments classified into this subgroup of the promotion variable.

*“Why didn’t you disclose the cod? I’ve even bought it.”*

*“Why isn’t there anything about cod?? That’s interesting.”*

Finally, end consumers also use social media to request improvements to the manufacturers’ websites. Even when manufacturers do not market their products directly to end customers, they can use their websites to promote products and engage in dialogues with customers. Websites can serve as a tool to support consumers’ journeys, bringing them closer to making a purchase. The reverse is also true: browsing the site is not pleasant, and inviting customers can cause frustration and alienate them. As noted in the comments below, consumers show a desire for sites that are more intuitive, easier to use, and provide the information that they seek. On the other hand, there are consumers who await responses from the company in exchange for their time and the provision of information that safeguards the maintenance of the relationship that has begun [19].

*“The site is confusing; I want to see the prices of the products “ “Where can I access the nutritional tables for your products? I couldn’t find them on the site (the one there with a TERRIBLE format!), nor here on your Instagram. Does anyone know where it is? Man, any little company has this information on its website, along with photos of the products for sale. The way it is, it’s impossible to recommend products to anyone who’s interested in them.” “I just think the site should be easier to use.”*

### 3.5. Content Generated by the Use of Social Media—Insights

The insights provided by examining the content generated on social media can aid manufacturers regarding the expected benefits and help marketers to direct more proactive strategies for companies producing plant-based alternative meat products. Based on the survey’s main findings, Table 8 presents the expected benefits by consumers and their insights and justifications, all supported by the four strategic variables of the marketing mix: product, price, place, and promotion.

When identifying consumers’ expected benefits, actions are proposed that plant-based alternative meat products manufacturers can implement to meet the expectations of final consumers. The proposed actions are classified according to the 4Ps of the marketing mix and represent possible responses to the desires expressed by consumers in their online interactions with companies. After all, the objective of identifying expected benefits by consumers is to obtain information to help marketers in their decision making to adopt more cost-effective strategies in this market niche.

Thus, from a practical and managerial point of view, this research can direct marketers of companies that manufacture plant-based meat analogs to (i) recognize the potential of using social media as a tool to improve the company’s engagement with its end consumers and aid the co-creation of value by providing the expected benefits; (ii) recognize critical points in responding to the expected benefits by consumers; and (iii) deliver more value to customers through marketing actions that stem from insights gleaned from analyzing the content generated by the consumers themselves. From a theoretical point of view, this research fills a gap in the literature by investigating the potential for the use of social media by manufacturers of plant-based alternative meat products regarding the goals of end consumers. Furthermore, since the results demonstrate the usefulness of social media for this purpose, the present study may encourage further research on social media analysis to understand the expected benefits by consumers in the plant-based meat alternatives market.

## 4. Final Considerations

Increasingly, food industries need to be aware of the benefits expected by consumers who want to reduce meat consumption. To conquer these consumers, companies must pay attention to the categories of expected benefits analyzed within a proposal of evidence from the marketing mix’s perspective to help consumers transition to a more plant-based meat alternatives diet. The plant-based alternative meat products market has attracted attention in recent years, as the demand for these products has grown worldwide. To meet the needs of this promising market, marketers must pay attention to the expected benefits of consumers and the insights that can be gleaned from comments posted on social media. Considering that people and companies increasingly interact on the Internet, this article proposed an investigation of the potential of the content analysis of comments posted on the Instagram social network of food companies that manufacture plant-based alternative meat products to understand the expected benefits by end consumers from the perspective of the classic marketing mix variables.

The content posted voluntarily by consumers was organized into 13 categories of expected benefits analyzed within a proposal of evidence from the perspective of the marketing mix. The results showed that, among the insights obtained, 63% were related to the place variable, 21% to the product variable, 11% to the price variable, and 5% to the promotion variable. The insights reinforce the notion that marketing mix variables are crucial factors for companies to make products available in the right place, in the right quantity, and at a fair price, in addition to engaging with consumers through social media. This research fills a gap in the literature related to communication through social media and can help marketers formulate more cost-effective strategies to reach their target audience.

As social media serve precisely to build relationships [41], manufacturers must provide consumers with answers, either through individualized feedback on each comment or through general compliance with the objectives that motivated the consumers’ interaction. The results of this study corroborate the research by Felix et al. [42], which highlights that companies can adopt two different types of strategies regarding the use of social media. “Explorer” companies use social media marketing in an integrated and bidirectional way to create and maintain reciprocal relationships with stakeholders. In contrast, “defensive” companies use social media only to convey information, providing their consumers with only standardized or non-standard responses in answer to their posts [42]. As proposed in this work, the purpose of using social media is to maintain a mutually beneficial relationship between manufacturers and final consumers to deliver their expected benefits. The response to consumers is certainly a point to which manufacturers should pay special attention. After all, meeting the desired goals will only occur if the information collected is used to improve services and exceed the expectations of the final consumer.

Finally, it is worth noting that this work makes new contributions to the field of plant-based alternative meat products by focusing on the expected benefits by consumers from the perspective of the marketing mix. Previous studies have explored aspects such as the environment, animal welfare, and health appeals, as well as dimensions related to consumer attitudes [5,28]. This article makes theoretical and managerial contributions to the field of plant-based alternative meat products. The results show the importance of analyzing content published on social media through the lens of the marketing mix for a better understanding of the expected benefits by consumers in the plant-based alternative meat products market. From a managerial point of view, this article presents findings that can guide the actions of manufacturers regarding the use of social media in exploring new insights through the lenses of the classic marketing mix. The purpose is to provide the expected benefits, conquering new consumers and markets for this specific niche. For future work, the categories identified through content analysis can help to conduct a survey with a broad sample of consumers to reinforce and expand the insights discovered in this work.

This article has limitations, as this study considered a limited time for collecting comments and focused on a single social network.

### Future Perspectives Research

Future research may also expand the number of companies evaluated, the number of social media, and the analysis time to test the consistency of the findings presented here. Furthermore, as this study focused on the expected benefits by customers, future research could identify strengths in terms of benefits already delivered to consumers. It is possible to note, for example, that despite consumers desiring improvements in terms of taste, many declare themselves satisfied with the products made available by the manufacturers. Although the present study did not exhaust such a comprehensive theme, the theoretical and practical implications can be used to support further research.

## Figures and Tables

**Table 1:** Inclusion and exclusion criteria for posts and comments.

Company Posts	
Inclusion Criteria	Exclusion Criteria
- Latest posts made by companies until the number of 300 comments collected on the profile of each manufacturer was reached so that updated data could be obtained and there was a balance between the number of comments collected from each manufacturer; - Posts that evidence the manufactured products or other aspects related to the companies.	- Posts that were intended to communicate partnerships with other companies to prevent the comments collected from reflecting the opinion of consumers about business partners and not about the companies analyzed; - Posts with targeted questions that could induce responses exclusively related to a particular subject, causing many comments on a single topic. For example, posts with questions such as “where would you like to find our products?” were avoided.
Comments	
Inclusion Criteria	Exclusion Criteria
- Primary comments; - Comments that contained text, since the methodology included textual analysis of the followers’ comments.	- Identical comments posted by the same follower in the same post to avoid duplication of data; - Secondary comments, that is, no comments were collected, as the objective was to capture consumers’ reaction to the companies’ posts and not the reaction of followers to the comments of other followers.

**Table 2:** Content analysis sample.

Sample Comments	Inductive Coding of Expected Benefits	Occurrences Number	Axial Coding—Marketing Mix
“I want the chicken of the future in FORTALEZA, for God’s sake.”	Coverage	111	Place (Distribution)
“I looked on the website, and it seems that it doesn’t sell anywhere in Brasília! Is that right?”	Distribution information	61	
“Incredible harvest here in Betim, Minas Gerais State. I don’t think ANYTHING!! They could sell directly on the website...”	Online channels	9	
“... Just one detail, the supermarkets still need to be supplied with more varieties. I live in Juazeiro do Norte, Ceará, and I always buy from Assaí there, but I only have kebabs and hamburgers. I want more he he e”	Assortment	6	
“Could you guys reduce the sodium content? I found it a little too salty.”	Nutritional, flavor, or texture improvements	24	Product
“It tastes like plastic (image). I made it with tomato sauce to try to change the taste, but even so, it was still horrible.”	Nutrition, ingredient, or preparation information	19	
“I really liked it, I just thought the ‘chicken’ was a bit rubbery, but I really liked the flavor!!!”	New products	17	
“But where does the chicken flavor come from??”	Availability of information	3	
“Now you just have to bring the price down so that everyone can have access to veganism... But I’m really happy that all of this stuff exists. I can’t wait to try it.”	Price reduction	28	Price
“How much do the sausages cost?”	Price information	5	
“Future Farm, don’t be a wet blanket, give me some freebies” “I just wanted to be sponsored by you, I’ll take care of the rest.”	Freebies, gifts, or sponsorship	9	Promotion
““Why don’t they advertise Cod???””	Greater disclosure	3	
“The site is confusing; I want to see the prices of the products.”	Website improvements	3	

Source: Instagram and research data.

**Table 3:** Results of the data analysis.

Category No.	Expected Benefits	Occurrences Number	Classic Marketing Mix Variables	Total
1	Coverage	111	Place (distribution)	187
2	Distribution information	61		
3	Online channels	9		
4	Assortment	6		
5	Nutritional, flavor, or texture improvements	24	Product	63
6	Nutrition, ingredients, or preparation information	19		
7	New products	17		
8	Availability of information	3		
9	Price reduction	28	Price	33
10	Prince information	5		
11	Freebies, gifts, or sponsorship	9	Promotion	15
12	Greater dissemination of products	3		
13	Website improvements	3		

**Table 4:** Subgroups of comments classified into the distribution pillar.

Insight No.	Expected Benefits	Occurrences Number	Variable	Total
1	Coverage	111	Place	187
2	Distribution information	61		
3	Online channels	9		
4	Assortment	6		

**Table 5:** Subgroup of products classified into the product variable.

Insight No.	Expected Benefits	Occurrences Number	Variable	Total
5	Nutritional, flavor, or texture improvements	24	Product	63
6	Nutrition, ingredient, or preparation information	19		
7	New products	17		
8	Information on availability	3		

**Table 6:** Subgroup of comments classified into the price variable.

Insight No.	Expected Benefits	Occurrences Number	Variable	Total
9	Price reduction	28	Price	33
10	Price information	5		

**Table 7:** Subgroups of comments ranked by the promotion variable.

Insight No.	Expected Benefits	Occurrences Number	Variable	Total
11	Freebies, gifts, or sponsorship	9	Promotion	15
12	Greater dissemination of products	3		
13	Website improvements	3		

**Table 8:** Benefits requested by consumers, insights, and justifications.

4Ps	Requested Benefits	Insights	Justification
Place	Coverage	- Distribute your products to cover the entire market.	Consumers want to easily find the products at points of sale in the regions close to their homes, especially at retail outlets where they make their purchases.
	Distribution information	- Provide customers with information regarding product availability; - Frequent communication with customers regarding where this information is available.	Customers want information on the points of sale where advertised products are available to facilitate their relationship and, consequently, their purchase process.
	Assortment	- Offer customers a variety of options on partner retail channels.	Customers do not wish to find only one or two kinds of products at the outlets they frequent, but rather a variety of products. For them, the advertised product has to be a product available for purchase.
Product	Nutritional, flavor, or texture improvements	- Improve your products constantly, both nutritionally and in terms of taste and texture.	Even if the products offered are already appreciated by consumers, improvements can still be made to them.
	Nutrition, ingredient, or preparation information	- Provide customers with nutritional information and information on the ingredients that make up the products in easily accessible locations;	Today’s consumers are increasingly well informed. They want to know what they are paying for and what they are eating. Therefore, providing clear information on the composition of the products is very important.
	New products	- Inform customers how they can prepare products in different ways. Do not allow them to purchase a product without knowing how to use it.	Customers also want to know every option open to them with regard to preparing products.
	Information on availability	- Develop new products such as cheese, butter, ham, and turkey to increase the variety of products offered.	Consumers want to find a wider variety of animal-like products. This can be an important growth opportunity for manufacturers.
Price	Price reduction	- Offer customers more attractive prices.	Do not allow costs to outweigh the potential benefits in customers’ views before they have even tried your product.
	Price information	- Inform customers of prices charged in easily accessible locations; - Offer customers information to justify the prices charged.	Customers want to know the prices of products even before going to the point of purchase. They also want to understand the reasons for the prices that are charged.
Promotion	Greater dissemination of products	- Widely advertise your products.	Customers want to receive information about products before finding them at the point of sale.
	Website improvements	- Offer customers a user-friendly website with the information they seek.	Meat substitutes are also aligned with a cause, and product promotion can help promote the cause, which can appeal to committed consumers.
